# Differential expression analysis of lncRNA and mRNA in ovarian tissues of Pishan Red Sheep and Hu Sheep with distinct genotypes during estrus

**DOI:** 10.3389/fvets.2025.1614599

**Published:** 2025-09-24

**Authors:** Aisima Muhetaer, Gao Gong, Yaoyang Ye, Ayipare Kuxitaer, Mengting Zhu, Qifa Li, Xing Du, Yiming Sulaiman

**Affiliations:** ^1^College of Animal Science, Xinjiang Agricultural University, Ürümqi, China; ^2^College of Animal Science and Technology, Nanjing Agricultural University, Nanjing, China

**Keywords:** Pishan Red Sheep, Hu Sheep, ovary, transcriptome, reproductive performance

## Abstract

Pishan Red Sheep and Hu Sheep are sheep breeds with exceptional reproductive characteristics. To investigate the similarities and differences in the expression of reproduction-related genes between these two breeds, this study utilized transcriptome sequencing to identify differentially expressed lncRNAs and mRNAs in ovarian tissues during estrus in Hu Sheep and Pishan Red Sheep carrying *FecB^B+^* and *FecB^++^* genotypes. Furthermore, we explored their potential impacts on fertility. Transcriptome sequencing of ovarian tissues generated 204.58 Gb of clean data. Bioinformatics analysis identified 34,651 lncRNAs, with differential expression analysis revealing 1,481 differentially expressed mRNAs and 698 differentially expressed lncRNAs. Differentially expressed RNAs associated with reproductive performance trends were screened through expression trend analysis. Functional enrichment analysis of target genes for these mRNAs and lncRNAs revealed significant enrichment in KEGG pathways such as “Cytokine-cytokine receptor interaction,” “Hippo signaling pathway” and “MAPK signaling pathway” Key candidate mRNAs were identified, including *GDF9*, *GRIA4*, *HOXC9*, *HOXD3*, *MAPK8IP3*, *AMH*, *ANGPT2*, *FGF14*, *MAPK8IP1*, *MMP9*, and *BRINP3*. Additionally, critical regulatory relationships between lncRNAs and mRNAs were uncovered. For example, *MSTRG.61044.1* exhibited high expression in *FecB^++^* genotype Pishan Red Sheep and may act as a hub regulator in follicular selection and hormonal responses by cis-regulating *MAPK8IP1* and trans-regulating *AMH*, *CCL25*, *MSTRG.23016.1* may regulate genes such as *MMP9* and *ANGPT2*, potentially participating in the modulation of the ovarian tissue remodeling microenvironment. In contrast, *MSTRG.15154.3* cis-regulates *ERBB4* to modulate the granulosa cell proliferation and differentiation process. The specifically highly expressed *MSTRG.2677.1* in Hu Sheep may be involved in maintaining ovarian stromal cell homeostasis through trans-regulation of *HGF* and *BRINP3*, *MSTRG.27015.1* and *MSTRG.60286.1* target *MAPK8IP3* and *PPP3CB* respectively, suggesting their potential roles in cell cycle regulation and oocyte maturation. These findings provide important molecular mechanisms and potential regulatory targets for improving reproductive performance in sheep.

## 1 Introduction

Pishan Red Sheep and Hu Sheep, as two important indigenous sheep breeds in China, have attracted significant attention due to their distinctive reproductive characteristics. Pishan Red Sheep are particularly renowned for their prolificacy, with notable differences in the expression of reproduction-associated genes observed between different lambing types (1). ChengLong et al. (1) demonstrated that in extreme environments, the population structure and genetic diversity of Pishan Red Sheep significantly influence their reproductive performance, with their prolific lambing traits being particularly prominent. Hu Sheep, characterized by their “year-round estrus and high prolificacy,” serve as an ideal model for studying the mechanisms underlying high fecundity (2). Relevant studies indicate that Hu Sheep, as a prolific sheep breed, can achieve a lambing rate exceeding 200% under optimal feeding conditions (3).

The litter size trait in mammals is governed by an integrated reproductive system, representing a complex quantitative trait regulated by multiple factors including genetics, environmental conditions, and hormonal levels (4). In sheep, litter size exhibits relatively low heritability, yet selective breeding through conventional methods can progressively enhance this trait and improve overall flock reproductive performance (5). With advancements in biotechnology, marker-assisted selection (MAS) has provided new opportunities to enhance selection efficiency for improving litter size in sheep (6). Long non-coding RNAs (lncRNAs) play critical regulatory roles in ovarian function and estrous cycle regulation in sheep (7). lncRNAs regulate gene expression through multiple mechanisms, including miRNA interactions (8), modulation of mRNA stability and translation efficiency (9), and epigenetic modification-mediated gene regulation (10). During the estrous cycle in sheep, the lncRNA expression profiles in ovarian tissues undergo dynamic changes, which may be functionally associated with follicular maturation, ovulation, and corpus luteum formation (11).

Litter size in sheep is regulated by the integrated ovarian reproductive system through diverse physiological and molecular mechanisms, including sex hormone secretion, follicular development and maturation, as well as lncRNA-mediated expression control of key genes (6). In sheep reproduction, the ovaries serve as one of the pivotal organs governing litter size (12). Through the secretion of sex hormones (e.g., estrogen and progesterone) and oocyte production, the ovaries regulate the reproductive cycle (13). During the estrous cycle, ovarian follicles undergo developmental progression from primordial to mature stages, culminating in oocyte release during ovulation (14). This process is precisely regulated by the hypothalamic–pituitary-ovarian (HPO) axis, with gonadotropin-releasing hormone (GnRH), follicle-stimulating hormone (FSH), and luteinizing hormone (LH) playing pivotal roles (15).

Advances in high-throughput sequencing technologies have accelerated research on the functional roles of lncRNAs in ovarian regulation of sheep reproduction (11, 16). Sheep with different *FecB* genotypes exhibit significant variations in reproductive performance (1), where *FecB^B+^* carriers demonstrate superior litter size traits compared to *FecB^++^*genotypes. Studies demonstrate that *FecB^B+^* sheep exhibit significantly higher litter sizes (1.453 ± 0.063) compared to *FecB^++^* genotypes (1.125 ± 0.059) (17). Existing studies have identified extensive differentially expressed genes (DEGs) across multiple tissues and blood transcriptomes among sheep with distinct FecB genotypes (18). Hu Sheep exhibit stable reproductive performance (19). As a representative prolific breed, their transcriptomic data provide critical references for comparative analyses (20). Current research on differential expression of lncRNAs and mRNAs in ovarian tissues during estrus between Pishan Red Sheep and Hu Sheep remains limited, particularly in comparative studies of different *FecB* genotypes. The regulatory networks and their underlying biological functions have yet to be systematically elucidated.

This study aims to delineate the regulatory mechanisms underlying ovarian physiology through comparative transcriptomic profiling of differentially expressed long non-coding RNAs (lncRNAs) and mRNAs in ovarian tissues during the estrous cycle between Pishan Red Sheep and Hu Sheep. Through systematic integration of lncRNA-mRNA co-expression networks, this investigation elucidates their functional interplay in modulating ovarian homeostasis, thereby establishing a molecular framework for enhancing ovine reproductive efficiency and informing marker-assisted selection strategies in genetic improvement programs.

## 2 Materials and methods

### 2.1 Animals and sample collection

The experimental animals used in this study, Hu Sheep and Pishan Red Sheep, were provided by Xiyu Muyangren Agriculture and Animal Husbandry Co, Ltd. in Pishan County, Hotan Prefecture. Experimental ewes were selected based on lambing records under uniform feeding conditions. The cohort comprised Hu Sheep (*FecB^BB^*), heterozygous *FecB^B+^* Pishan Red Sheep, and wild-type *FecB^++^* Pishan Red Sheep, all being healthy, non-related nulliparous ewes aged 7–8 months with consistent body conformation, the number of animals per group was *n* = 4.

### 2.2 Estrus synchronization and experimental grouping

Estrus synchronization was performed on all 18 selected ewes using a single prostaglandin F2α (PGF2α)-based protocol. The luteal phase was initially confirmed through visual assessment of estrus signs. Each ewe received an intramuscular injection of 0.1 mg PGF2α. At 48 h post-injection, estrus behavior was detected using the ram exposure method to determine optimal timing for sample collection.

### 2.3 Ovarian tissue collection and experimental grouping

Four estrous Pishan Red Sheep with *FecB^B+^* genotype (Group A), four with *FecB^++^* genotype (Group B), and four Hu Sheep (Group C) were selected and humanely slaughtered. Ovarian tissue samples were promptly collected, and tissues surrounding the ovaries were removed. The samples were rinsed with phosphate-buffered saline (PBS), transferred to RNase-free cryotubes, and immediately snap-frozen in liquid nitrogen. All animal experimental procedures strictly adhered to the Institutional Animal Care and Use Guidelines of Xinjiang Agricultural University.

### 2.4 Experimental materials

The experiment employed the following instrumentation to meet operational requirements across all experimental stages: PCR system (Life Technologies), dry bath incubator (TIANGEN), mini centrifuge (TIANGEN), vortex mixer (Vortex-Genie 2, Scientific Industries), adjustable pipettes (RAININ), magnetic separation rack (Life Technologies), tube rotator (QLinbeier), Qsep-400 automated electrophoresis system (BiOptic Inc.), and Qubit 3.0 Fluorometer (Thermo Fisher Scientific).

### 2.5 RNA extraction and library construction

Total RNA was extracted from ovine ovarian tissues using TRIzol reagent (Thermo Fisher Scientific, USA). RNA concentration was measured with a NanoDrop 2000 spectrophotometer (Thermo Fisher Scientific, USA), while RNA integrity was assessed using an Agilent 2,100 Bioanalyzer (Agilent Technologies, USA). Library quality control was performed with the Qsep-400 system (BiOptic Inc., Taiwan, China). Qualified libraries were subsequently sequenced on the NovaSeq X Plus platform (Illumina, USA) with 150 bp paired-end reads. The RNA-Seq data of Pishan Red Sheep and Hu Sheep have been deposited in the Sequence Read Archive (SRA) database of the National Center for Biotechnology Information (NCBI) under BioProject accession number PRJNA1226111.

### 2.6 Screening of differentially expressed mRNAs and lncRNAs in ovarian tissues

Following sequencing, the data were analyzed using the bioinformatics pipeline provided by the BMKCloud platform^1^ from Biomarker Technologies. The FASTQ data obtained from high-throughput sequencing were analyzed to predict and identify multiple RNA species, followed by quantitative profiling of distinct RNA categories through alignment with established RNA annotation databases. In this study, differentially expressed genes (DEGs) and lncRNAs were screened using stringent thresholds of |Fold Change| ≥ 1.5 and *p*-value <0.01. Differential comparison groups: A-L vs. B-L, A-L vs. C-L, B-L vs. C-L. Target genes of lncRNAs were predicted using two distinct approaches. The first method focused on cis-regulatory mechanisms, identifying adjacent protein-coding genes located within a 100 kb genomic window upstream/downstream of lncRNA loci as potential targets, based on the positional relationship between lncRNAs and their neighboring genes. The second approach employed trans-regulatory prediction by analyzing inter-sample expression correlation between lncRNAs and mRNAs, identifying potential target genes through co-expression patterns.

### 2.7 Trend analysis

The differentially expressed lncRNAs and mRNAs identified were further analyzed to investigate their expression dynamics across different genotypes. Gene expression dynamics were visualized through trend analysis plots to delineate specific expression pattern alterations.

### 2.8 Enrichment analysis

The Gene Ontology (GO) database, established in 2000 by the Gene Ontology Consortium, constitutes a cross-species standardized annotation framework for genes and their products, structured into three orthogonal categories: Biological Process (BP), Molecular Function (MF), and Cellular Component (CC). Following gene annotation, functional categorization of differentially expressed genes (DEGs) was conducted at the second-level Gene Ontology (GO) classification. Enrichment analysis of DEG sets from each comparison group was performed using the hypergeometric test implemented in the clusterProfiler R package (v4.0.5), with results visualized as bar plots. A lower *q*-value indicates greater statistical significance of the enriched functional pathways, thereby facilitating the functional inference of candidate genes. Pathway enrichment analysis of differentially expressed genes was conducted using the KEGG (Kyoto Encyclopedia of Genes and Genomes) database. KEGG pathways were utilized as ontological units, and the hypergeometric test was applied to identify significantly enriched pathways (adjusted *p*-value < 0.01) by comparing against the genomic background. Concurrently, KEGG pathway annotation results for both target genes of differentially expressed lncRNAs and mRNAs were systematically categorized according to KEGG pathway classifications, thereby identifying key biochemical metabolic and signal transduction pathways in which these genes are functionally implicated. The present investigation primarily centers on biological pathways associated with reproductive physiology and ovarian functional regulation.

### 2.9 Screening of key mRNAs and lncRNAs

This study implemented an integrated methodology combining systematic literature review and multi-omics database mining to identify functionally pivotal genes associated with ovine reproductive performance. The selection criteria prioritized core constituents within key reproductive pathways, including the estrogen signaling pathway, MAP kinase cascade, cytokine-cytokine receptor interaction, and Hippo signaling pathway. Corresponding nucleotide sequences and spatiotemporal expression profiles were retrieved from the NCBI GenBank and GEO database. Differential expression analysis of mRNA and lncRNA was conducted using DESeq2 and edgeR packages on ovarian tissue samples from *FecB^++^*-genotype Pishan Red Sheep, *FecB^B+^*-genotype Pishan Red Sheep, and Hu Sheep, based on high-throughput sequencing data to identify genes with statistically significant expression changes.

### 2.10 Integrated analysis of hub mRNAs and key lncRNAs

During the integrated analysis of hub mRNAs and key lncRNAs, significantly differentially expressed mRNAs and lncRNAs were first identified. Subsequently, a cross-correlation heatmap was constructed to visualize expression covariation patterns between these two RNA classes.

### 2.11 Real-time quantitative fluorescence

To validate the expression levels of key candidate genes identified from screening, we performed qRT-PCR analysis on additional ovine ovarian tissue samples, thereby confirming the reliability of both sequencing and bioinformatics results. Using *β*-Actin as the internal reference, the relative gene expression levels were calculated for each sample and group. The processed data were organized in Excel and subsequently analyzed for visualization using Origin 2024 software. Primer sequences are provided in Table 1. Primers were designed using Premier 6 (Premier Biosoft, USA) and synthesized by Biomarker Technologies (Beijing, China). cDNA was reverse transcribed from total RNA, followed by RT-PCR amplification. Quantitative PCR was performed under the following conditions: initial denaturation at 95 °C for 3 min; 39 cycles of 95 °C for 10 s (denaturation), 60 °C for 30 s (annealing/extension) with plate reading; followed by a melting curve analysis (60–95 °C, increment of 1 °C per cycle, 4 s hold time).

**Table 1:** List of PCR primer sequences.

Gene Name	Sequence (5′ → 3′)	Product (bp)
*β-Actin*	F:CGTGCGTGACATCAAAGAGAA R:AACCGCTCGTTGCCAATAGT	141
*MMP9*	F:GCACGCACGACATCTTTCAG R:AGGAGGTCGAAGGTCACGTA	115
*AMH*	F:GCTCATCCCCGAGACATACC R:GATGAGGAGCTTGCCTGTGT	181
*MAPK8IP3*	F:CAACACCGACTCCCTGTACC R:ACGCACTGAGAACTCCCCTA	100
*ANGPT2*	F:AGCACGAAGGATGCAGACAA R:CCATTCAGGTTGGAGGGACC	101
*MSTRG.60286.1*(lncRNA)	F:GGCACAGCATTTTTAGTGCCT R:CACCCAGTGGACCTTCTTCC	165

## 3 Results

### 3.1 Total RNA quality assessment

The detection conclusions were based on Biomarker Beijing’s sequencing sample detection standard requirements and constituted a comprehensive evaluation of the tested samples. The samples with Test Result A meet the quality requirements for library construction, and the total quantity is sufficient for two or more standard library preparations. RNA quantification via NanoDrop demonstrated a concentration range of 98.9–652.8 ng/μL with total yields ranging from 1.48 to 9.79 μg. RNA Integrity Numbers (RIN) were distributed between 6.1 and 8.8, while 28S/18S rRNA ratios spanned 1.0 to 4.03. Detailed mRNA extraction parameters are presented in Table 2.

**Table 2:** Information on sample mRNA extraction.

Breeds	Detection results	Nanodrop detection concentration (ng/ul)	Volume (ul)	Total quantity (ug)	RIN value	28S/18S
A1-L	A	253	15	3.80	7.7	3.13
A4-L	A	517.2	15	7.76	6.8	1
A2-L	A	652.8	15	9.79	8.8	4.03
A3-L	A	302.3	15	4.53	7	3.18
B1-L	A	481.4	15	7.22	6.2	1.37
B2-L	A	189.1	15	2.84	7.2	3.03
B3-L	A	374.1	15	5.61	6.5	1.75
B4-L	A	493.9	15	7.41	6.6	1.89
C1-L	A	119.9	15	1.80	6.1	1.59
C2-L	A	455.9	15	6.84	6.7	1.74
C3-L	A	294.9	15	4.42	6.6	2.81
C4-L	A	98.9	15	1.48	6.8	1.96

### 3.2 Quality and alignment information of the test data

Strand-specific RNA sequencing libraries were constructed and sequenced on the NovaSeq X Plus platform (Illumina). Following stringent quality control, a total of 204.58 Gb clean data were obtained with Q20 > 97.45% and Q30 > 93.31%, while maintaining low N ratios and stable GC content, indicating high-quality sequencing outputs (detailed metrics in Table 3). Reference genome alignment statistics revealed mapping rates ranging from 81.40 to 91.19% against the *Ovis aries* reference genome assembly (Ovis_aries.GCF_000298735.2_Oar_v4.0.genome.fa). Complete alignment results are tabulated in Table 4.

**Table 3:** Assessment statistics of sample sequencing data.

Breeds	Sample ID	Read sum	Base sum	GC (%)	*N* (%)	Q20 (%)	Q30 (%)
*FecB^B+^* Type Pishan Red Sheep	A1-L	55,147,707	16,507,803,237	47.07	0.02	97.76	94.06
	A2-L	56,231,640	16,824,877,240	48.12	0.02	97.84	94.25
	A3-L	55,010,326	16,452,282,004	48.72	0.02	97.65	93.98
	A4-L	57,751,857	17,264,327,636	46.37	0.01	98.06	94.74
*FecB^++^* Type Pishan Red Sheep	B1-L	55,715,053	16,671,403,429	48.69	0.02	97.48	93.64
	B2-L	55,891,691	16,702,518,965	49.71	0.02	97.60	93.82
	B3-L	57,305,418	17,145,581,750	47.05	0.02	97.87	94.35
	B4-L	57,646,787	17,257,333,335	47.01	0.02	97.80	94.08
Hu Sheep	C1-L	56,729,728	16,965,171,835	48.82	0.01	97.45	93.31
	C2-L	54,511,792	16,308,097,034	47.54	0.02	97.64	93.75
	C3-L	55,652,920	16,644,744,049	47.60	0.02	97.57	93.74
	C4-L	66,300,012	19,833,530,886	47.51	0.01	97.64	93.77

**Table 4:** Statistics of sequence alignment results of sample sequencing data with the selected reference genome.

Breeds	Sample ID	Total reads	Mapped reads	Uniq mapped reads	Multiple mapped reads
*FecB^B+^* Type Pishan Red Sheep	A1-L	110,295,414	100,249,721 (90.89%)	93,173,782 (84.48%)	7,075,939 (6.42%)
	A2-L	112,463,280	102,181,710 (90.86%)	92,945,700 (82.65%)	9,236,010 (8.21%)
	A3-L	110,020,652	93,974,246 (85.42%)	86,584,323 (78.70%)	7,389,923 (6.72%)
	A4-L	115,503,714	105,223,523 (91.10%)	98,986,052 (85.70%)	6,237,471 (5.40%)
*FecB^++^* Type Pishan Red Sheep	B1-L	111,430,106	99,336,048 (89.15%)	90,783,819 (81.47%)	8,552,229 (7.67%)
	B2-L	111,783,382	100,305,632 (89.73%)	90,254,939 (80.74%)	10,050,693 (8.99%)
	B3-L	114,610,836	104,509,269 (91.19%)	96,483,939 (84.18%)	8,025,330 (7.00%)
	B4-L	115,293,574	103,840,153 (90.07%)	98,081,800 (85.07%)	5,758,353 (4.99%)
Hu Sheep	C1-L	113,459,456	92,351,227 (81.40%)	86,999,334 (76.68%)	5,351,893 (4.72%)
	C2-L	109,023,584	99,324,537 (91.10%)	91,705,783 (84.12%)	7,618,754 (6.99%)
	C3-L	111,305,840	99,659,995 (89.54%)	91,075,550 (81.82%)	8,584,445 (7.71%)
	C4-L	132,600,024	119,959,119 (90.47%)	110,689,067 (83.48%)	9,270,052 (6.99%)

### 3.3 Comparative analysis of lncRNA and mRNA in sheep ovaries with different fecundity

**Figure 1:**
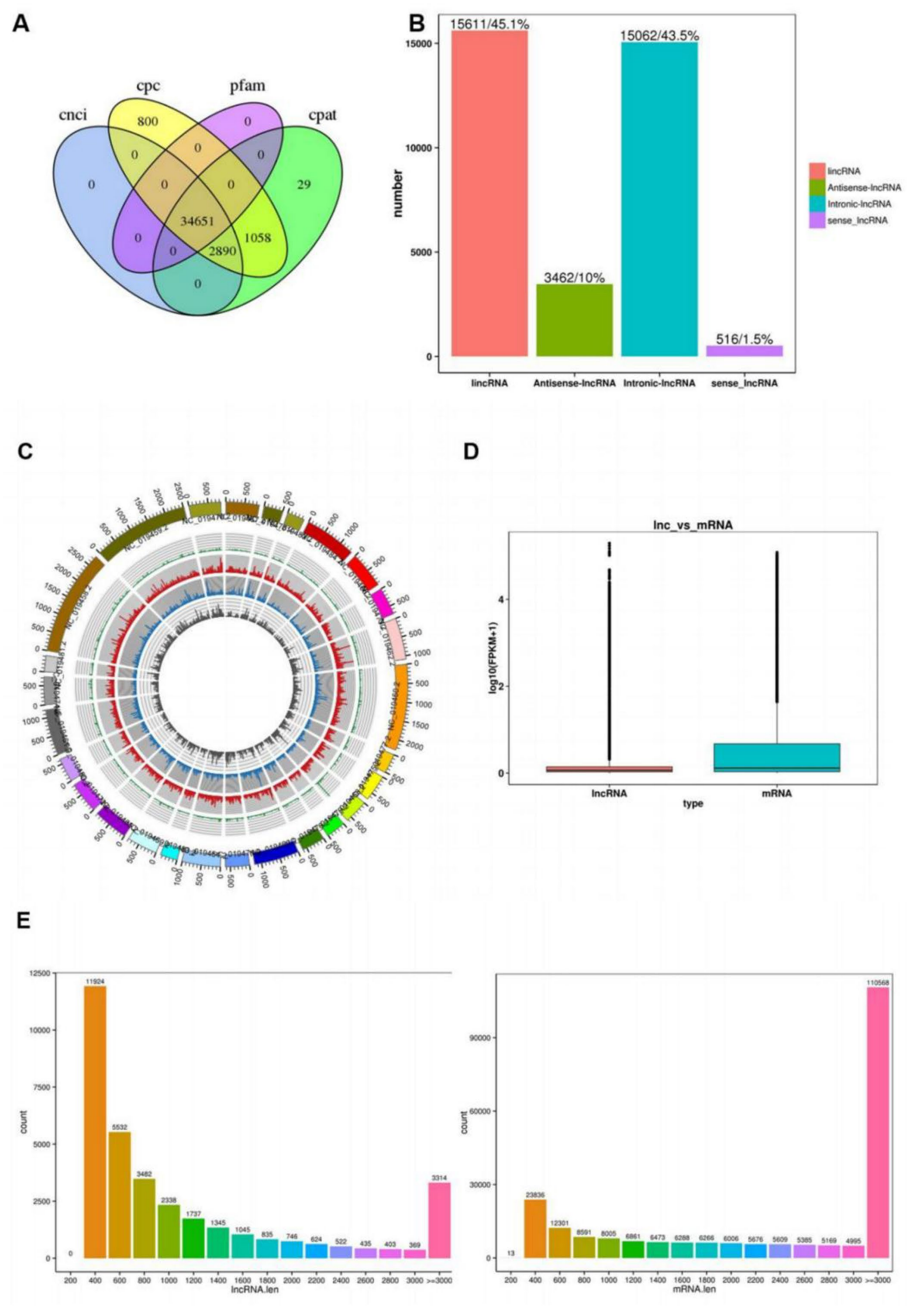
Identification of lncRNAs and mRNAs in ovaries of sheep with different fecundity. **(A)** Demonstrates the coding potential of lncRNAs predicted by four bioinformatics tools: CNCI, CPC, Pfam, and CPAT; **(B)** The *X*-axis represents four distinct types of lncRNAs, The *Y*-axis represents the number of corresponding lncRNAs; **(C)** The middle ring depicts the chromosomal distribution of differentially expressed mRNAs, while the inner ring represents the chromosomal distribution of differentially expressed lncRNAs. Within the rings, red and green denote upregulated and downregulated mRNAs, respectively; Yellow and blue indicate upregulated and downregulated lncRNAs, correspondingly; **(D)** Displays the expression levels of mRNAs and lncRNAs, The *Y*-axis represents log2-transformed FPKM (Fragments Per Kilobase per Million mapped reads) values; **(E)** Illustrates the transcript length distribution, The *Y*-axis indicates the number of corresponding lncRNAs.

The coding potential of putative lncRNAs was systematically assessed using four distinct computational tools: CNCI, CPC2, Pfam Scan, and CPAT. The intersection analysis of these predictions (Figure 1A) identified 34,651 high-confidence lncRNAs with conserved non-coding features. As depicted in Figure 1B, the composition of lncRNA classes was: intergenic lncRNAs (15,611, 45.1%), antisense lncRNAs (3,462, 10%), intronic lncRNAs (15,062, 43.5%), and sense lncRNAs (516, 1.5%). As illustrated in Figure 1C, the middle ring depicts the chromosomal distribution of differentially expressed mRNAs, while the inner ring represents the chromosomal distribution of differentially expressed lncRNAs. Within the rings, red and green denote upregulated and downregulated mRNAs, respectively; yellow and blue indicate upregulated and downregulated lncRNAs, correspondingly. As shown in Figure 1D, the expression levels of lncRNAs were consistently lower than mRNAs overall. Figure 1E illustrates the transcript length distribution, revealing that lncRNAs are primarily distributed between 200 and 400 bp, whereas mRNAs predominantly range from ≥3,000 bp.

### 3.4 Differential expression analysis of lncRNA and mRNA between different groups

**Figure 2:**
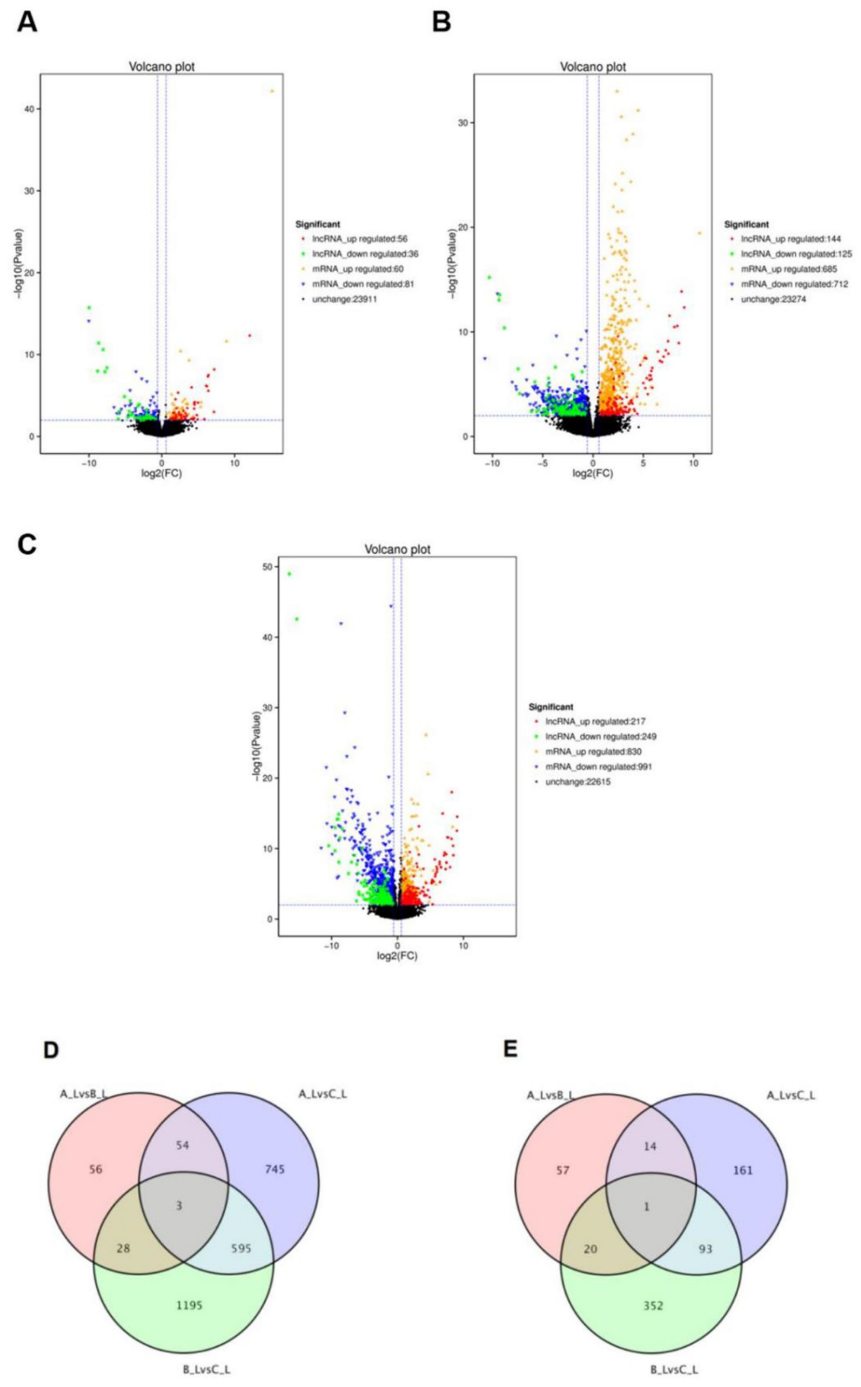
Differential expression of lncRNAs and mRNAs between different groups. **(A–C)** Volcano plots of differentially expressed mRNAs and lncRNAs for A-LvsB-L, A-LvsC-L and B-LvsC-L comparisons, respectively; **(D)** Venn diagram of differentially expressed mRNAs among A-LvsB-L, A-LvsC-L and B-LvsC-L groups; **(E)** Venn diagram of differentially expressed lncRNAs among A-LvsB-L, A-LvsC-L and B-LvsC-L groups.

Comparative analysis revealed distinct differential expression profiles across the three comparison groups: A-L vs. B-L (Figure 2A), A-L vs. C-L (Figure 2B), and B-L vs. C-L (Figure 2C). Specifically, 92 differentially expressed lncRNAs (56 upregulated, 36 downregulated), 269 lncRNAs (144 upregulated, 125 downregulated), and 466 lncRNAs (217 upregulated, 249 downregulated) were identified in the respective groups. Concurrently, 141 differentially expressed mRNAs (60 upregulated, 81 downregulated), 1,397 mRNAs (685 upregulated, 712 downregulated), and 1,821 mRNAs (830 upregulated, 991 downregulated) were detected in these comparisons. To comprehensively characterize the transcriptional changes in ovarian tissues during estrus, we identified differentially expressed long non-coding RNAs (lncRNAs) and messenger RNAs (mRNAs). The top five upregulated and downregulated lncRNAs and mRNAs are presented in Supplementary Tables 1 and 2, respectively. Venn analysis demonstrated that 1,481 shared differentially expressed mRNAs (Figure 2D) and 698 shared differentially expressed lncRNAs (Figure 2E) overlapped across all three groups.

### 3.5 Expression trends of mRNA and lncRNA

**Figure 3:**
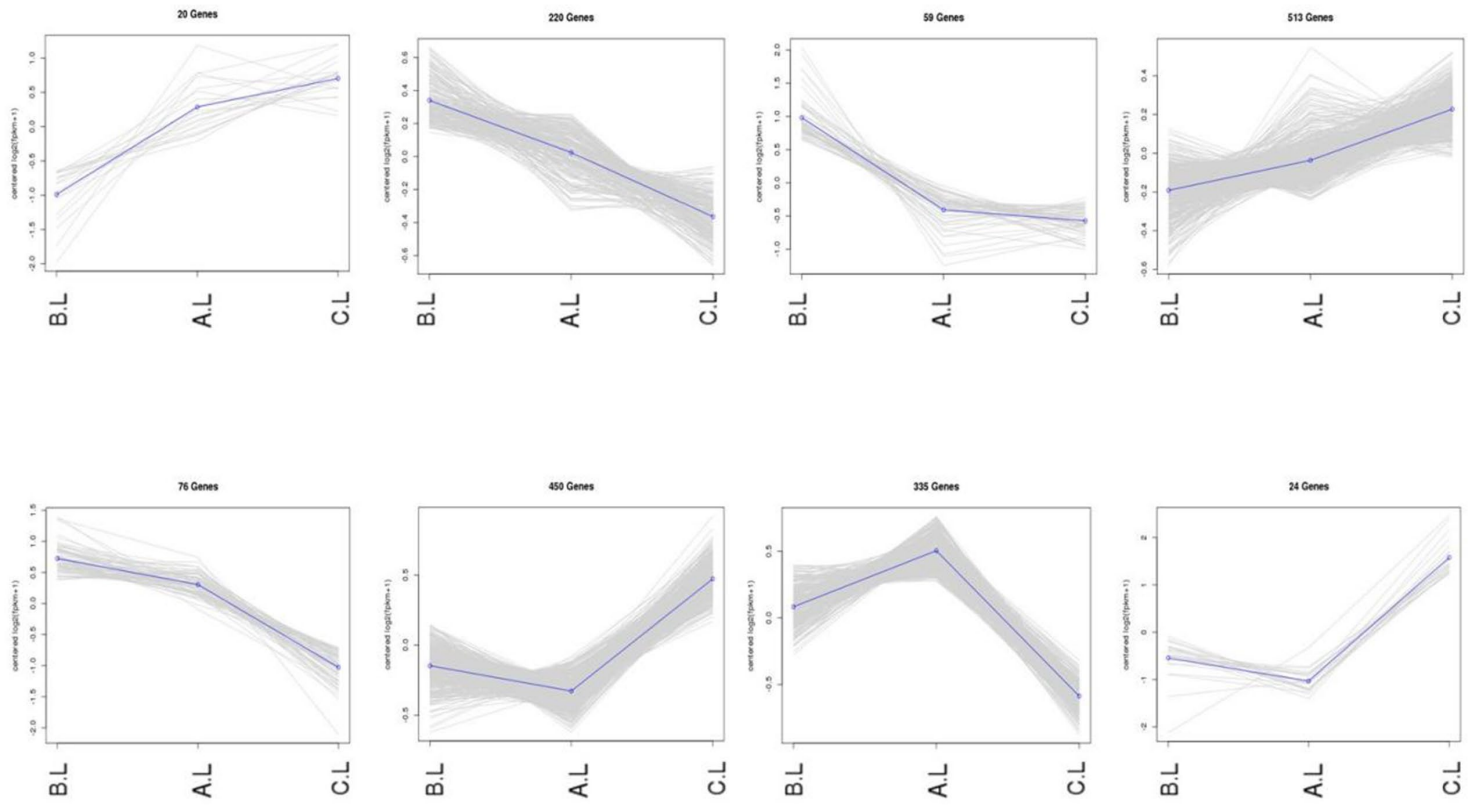
Co-expression trends of differentially expressed mRNA genes. The figure displays the variations in gene expression levels across distinct experimental conditions. In the trend analysis result figures, each panel is arranged in the order of *FecB^++^* genotype Pishan Red Sheep (Group B), *FecB^B+^* genotype (Group A), and Hu Sheep (Group C). The *Y*-axis represents mean-centered log2-transformed gene expression values.

**Figure 4:**
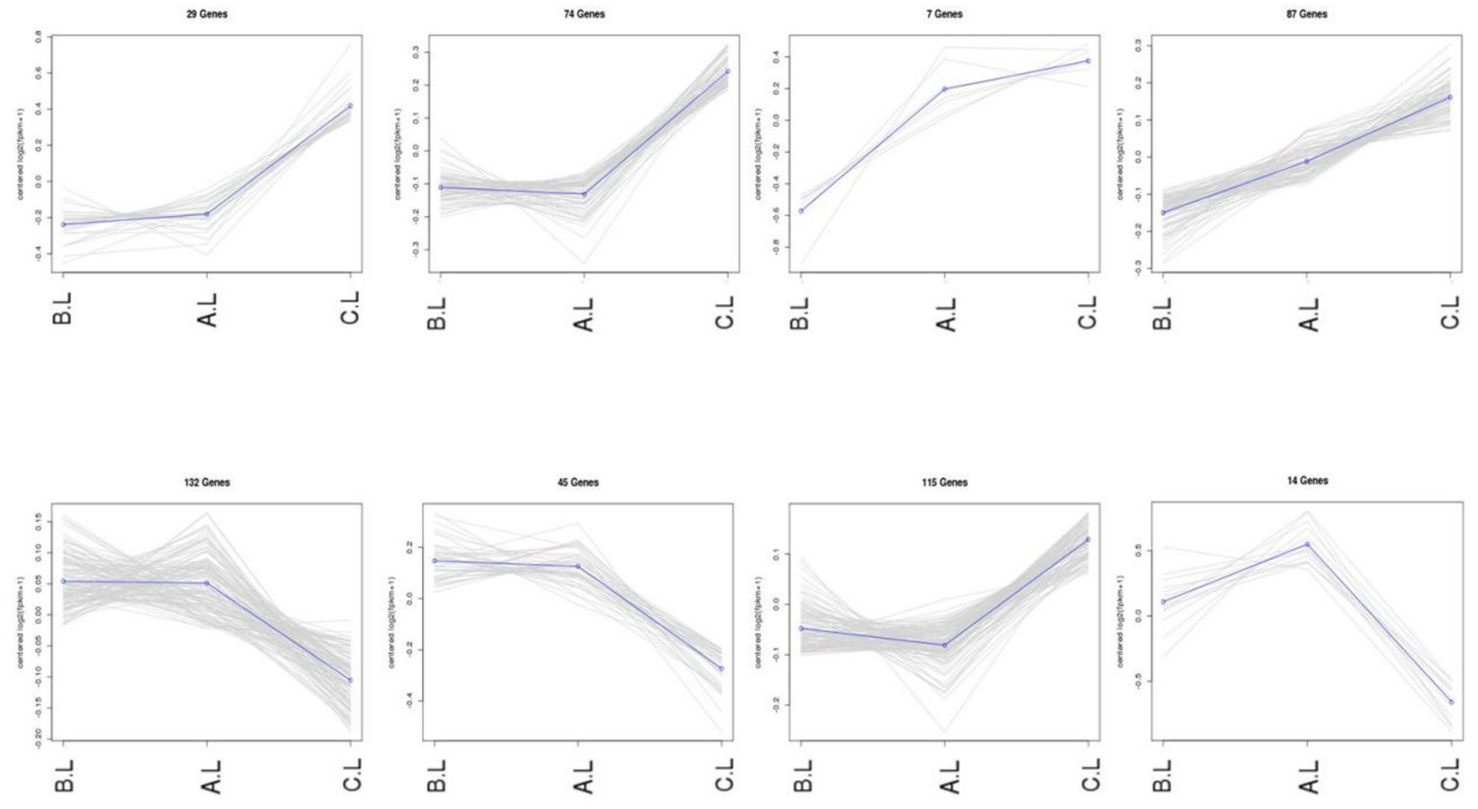
Co-expression trends of differentially expressed lncRNA genes. The figure displays the variations in gene expression levels across distinct experimental conditions. In the trend analysis result figures, each panel is arranged in the order of *FecB^++^* genotype Pishan Red Sheep (Group B), *FecB^B+^* genotype (Group A), and Hu Sheep (Group C). The *Y*-axis represents mean-centered log2-transformed gene expression values.

Expression trend analysis was performed on differentially expressed mRNAs and lncRNAs (Figures 3, 4). In the trend analysis result figures, each panel is arranged in the order of *FecB^++^*-genotype Pishan Red Sheep (Group B), *FecB^B+^*-genotype (Group A), and Hu Sheep (Group C). Clusters were selected based on the lambing performance patterns of these three types of sheep. The selection criteria were defined as follows: clusters exhibiting either consistently increasing or decreasing expression trends across all three groups, lower expression levels in Group B, but higher levels in Groups A and C, or higher expression levels in Group B, but lower levels in Groups A and C. This screening identified 888 differentially expressed mRNAs and 6,740 differentially expressed lncRNAs conforming to these expression trends.

### 3.6 Prediction of lncRNA target genes

LncRNAs orchestrate a broad spectrum of critical physiological processes through transcriptional and post-transcriptional regulation of target genes. Leveraging trend-based expression profiles, we performed systematic prediction of both cis-acting and trans-regulatory target genes for these prioritized lncRNAs. Cis-regulatory target genes were predicted by identifying protein-coding genes located within ±100 kb genomic regions flanking the lncRNAs using a Perl script, yielding 845 cis-target pairs. Furthermore, trans-regulatory targets were determined through Pearson correlation analysis of inter-sample co-expression patterns between lncRNAs and mRNAs, identifying 5,895 trans-target interactions.

### 3.7 Functional enrichment analysis of mRNA

**Figure 5:**
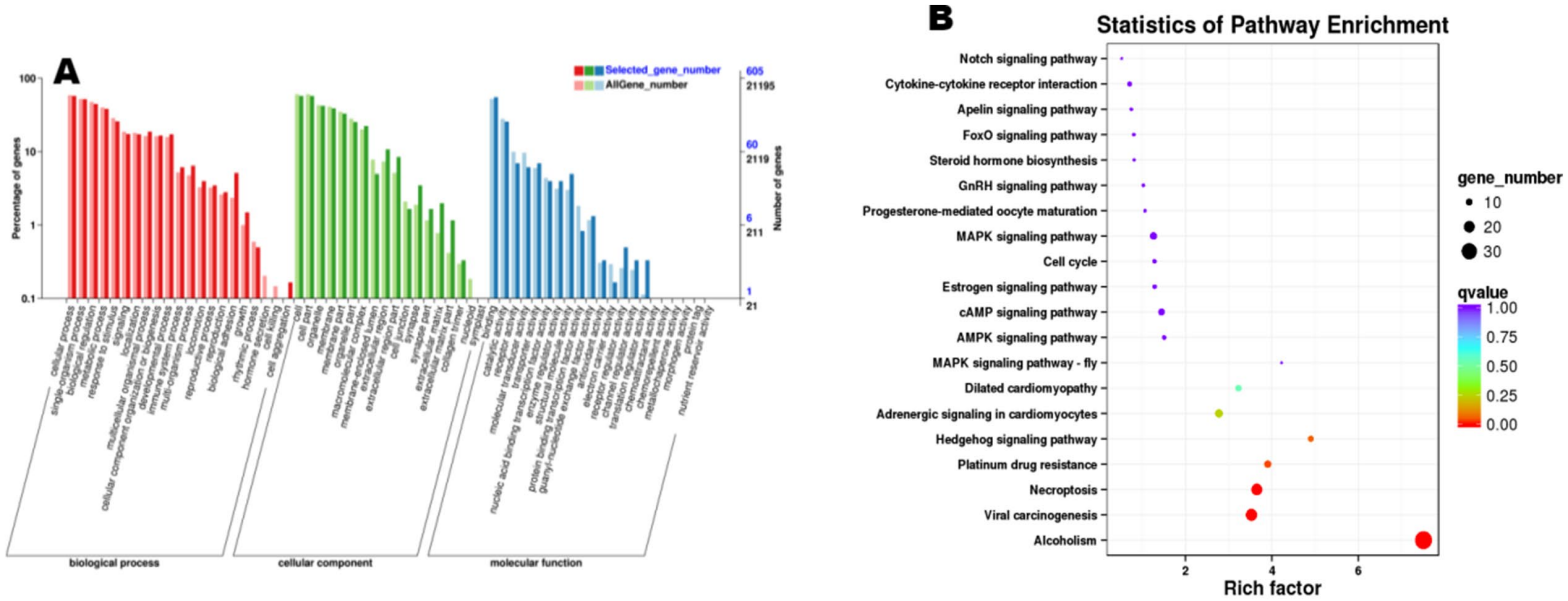
Functional enrichment analysis of differentially expressed mRNA. **(A)** The *X*-axis denotes Gene Ontology (GO) terms, while the *Y*-axis represents the number of genes. Distinct color codes distinguish major GO categories, with darker colors corresponding to upregulated genes and lighter colors indicating downregulated genes; **(B)** each circle represents a KEGG pathway. The *Y*-axis shows pathway names, and the *X*-axis corresponds to the Rich Factor (defined as the ratio of the proportion of differentially expressed genes annotated to a specific pathway to the proportion of all genes annotated to that pathway).

Functional enrichment analysis was performed on the 888 trending differentially expressed genes (DEGs), revealing significant enrichment of 60 GO terms and 285 KEGG pathways (Figure 5). Significant enrichments were observed in biological processes (Biological Process, BP), including key terms such as “cellular process” and “metabolic process.” Furthermore, KEGG pathway analysis revealed differential enrichment across multiple pathways, including cysteine and methionine metabolism, axon guidance, and Fc gamma receptor (FcγR)-mediated phagocytosis, among others. Notably, reproduction-associated pathways including progesterone-mediated oocyte maturation, estrogen signaling pathway, and gonadotropin-releasing hormone (GnRH) signaling pathway were prominently enriched in the ovarian mRNA enrichment analysis. These findings suggest that genes within these pathways may play pivotal roles in ovarian physiological activities and reproductive regulation during the estrus phase in sheep.

### 3.8 Functional enrichment analysis of ncRNA target genes

**Figure 6:**
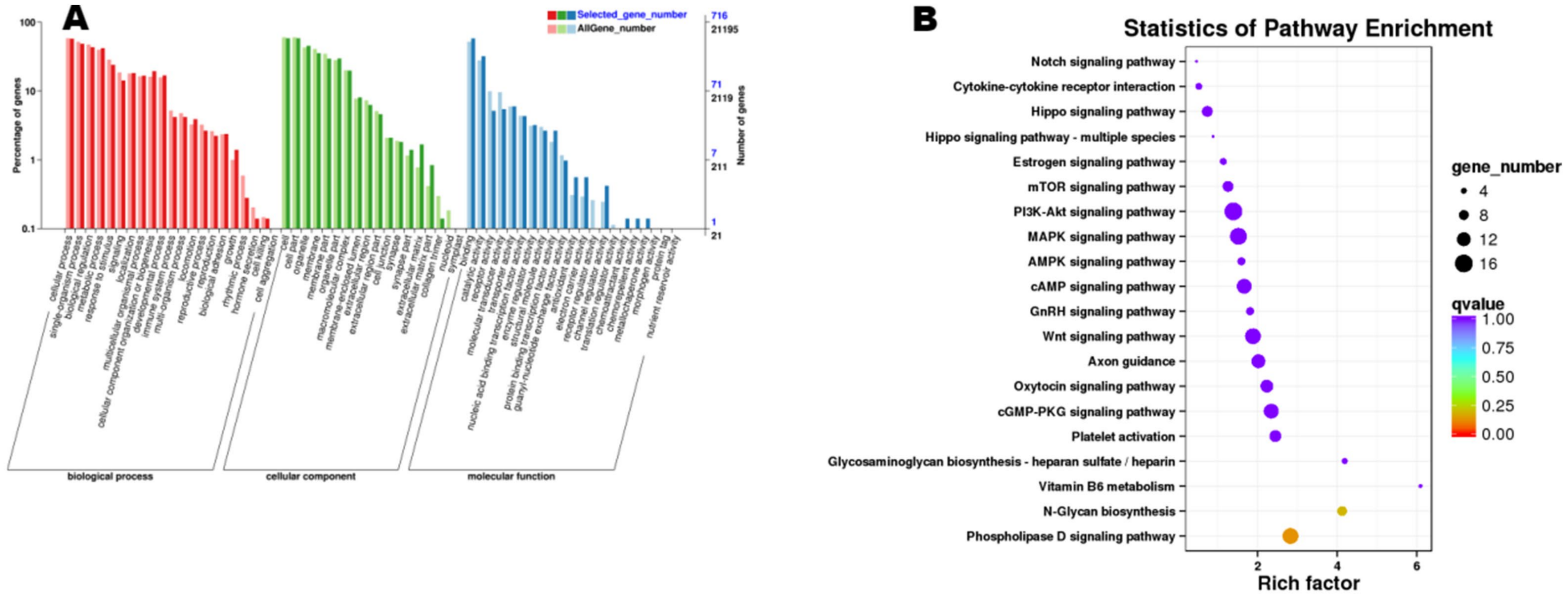
Functional enrichment analysis of cis-target genes of differential lncRNAs. **(A)** shows the GO enrichment analysis of cis-target genes, and **(B)** presents the results of KEGG analysis.

**Figure 7:**
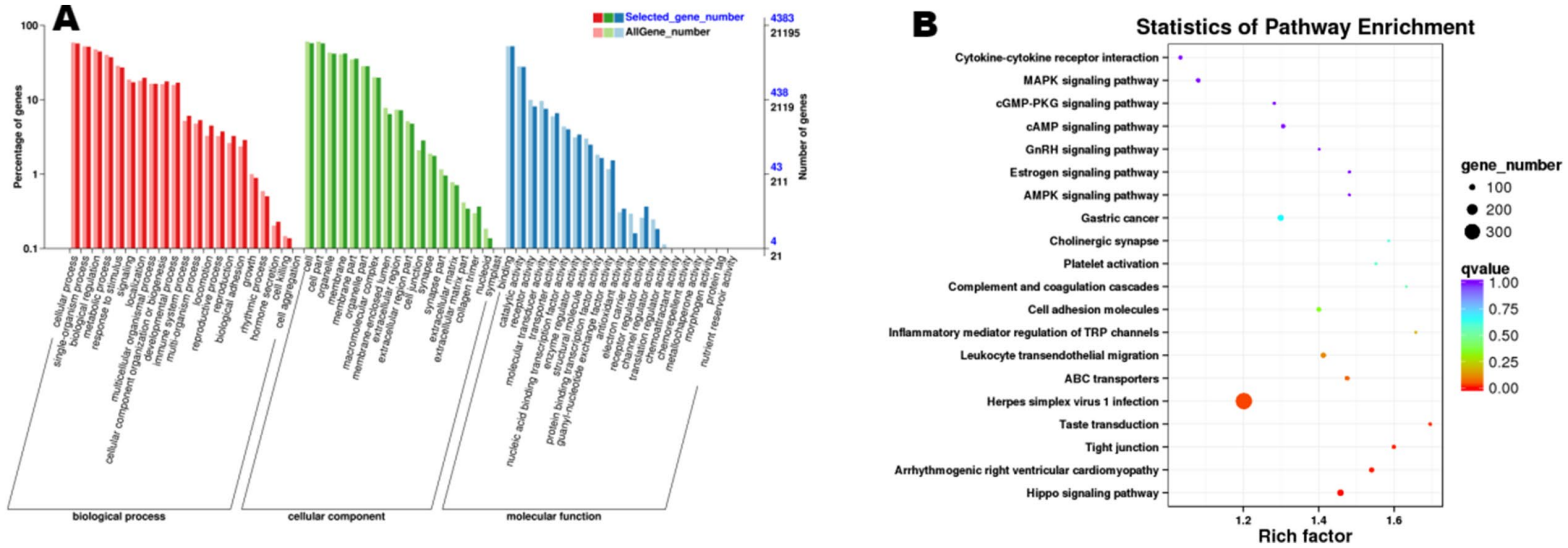
Functional enrichment analysis of trans-target genes of differential lncRNAs. **(A)** shows the GO enrichment analysis of trans-target genes, and **(B)** presents the results of KEGG analysis.

Functional enrichment analysis of the 845 cis-target genes and 5,859 trans-target genes predicted from the 6,704 trend-conforming lncRNAs revealed significant enrichments of 60 and 63 GO terms, and 289 and 330 KEGG pathways, respectively. GO enrichment analysis of cis-target genes (Figure 6) demonstrated significant enrichment in biological processes (BP), including “cellular process,” “single-organism process,” “biological regulation,” and “metabolic process,” indicating potential functional roles of these genes in diverse biological processes within ovarian tissues. KEGG pathway analysis of cis-target genes (Figure 6) revealed differential enrichment across multiple pathways, including “Cytokine-cytokine receptor interaction,” “Hippo signaling pathway,” and “Estrogen signaling pathway,” among others. GO enrichment analysis of trans-acting target genes (Figure 7) revealed that in the biological process category, entries such as “cellular process” and “single-organism process” exhibited relatively higher numbers of enriched lncRNA trans-acting target genes. This observation suggests that during physiological activities in ovarian tissues at the estrus phase, these biological processes may be significantly influenced by genes under trans-regulatory control of corresponding lncRNAs. KEGG pathway analysis (Figure 7) revealed significant enrichment of lncRNA trans-target genes in ovarian tissues for key pathways including “Cytokine-cytokine receptor interaction,” “MAPK signaling pathway,” and “cGMP-PKG signaling pathway.” These findings suggest these pathways may exert central regulatory functions in ovarian physiological processes during the estrous cycle. Furthermore, pathways including the “MAPK signaling pathway” and “cAMP signaling pathway” demonstrated consistent enrichment in both cis- and trans-target gene KEGG analyses, suggesting their potential roles in regulating signal transduction, cellular proliferation, and differentiation within ovarian tissues. The co-enrichment of these pathways further elucidates the multifaceted mechanisms by which lncRNAs orchestrate ovarian functional regulation.

### 3.9 Screening of potential functional mRNAs and lncRNAs involved in the reproductive process

Functional enrichment analysis identified a cohort of significantly expressed mRNAs and lncRNAs in ovarian tissues during the estrous cycle, particularly enriched in key KEGG pathways including “Cytokine-cytokine receptor interaction,” “Hippo signaling pathway,” and “MAPK signaling pathway.”

**Figure 8:**
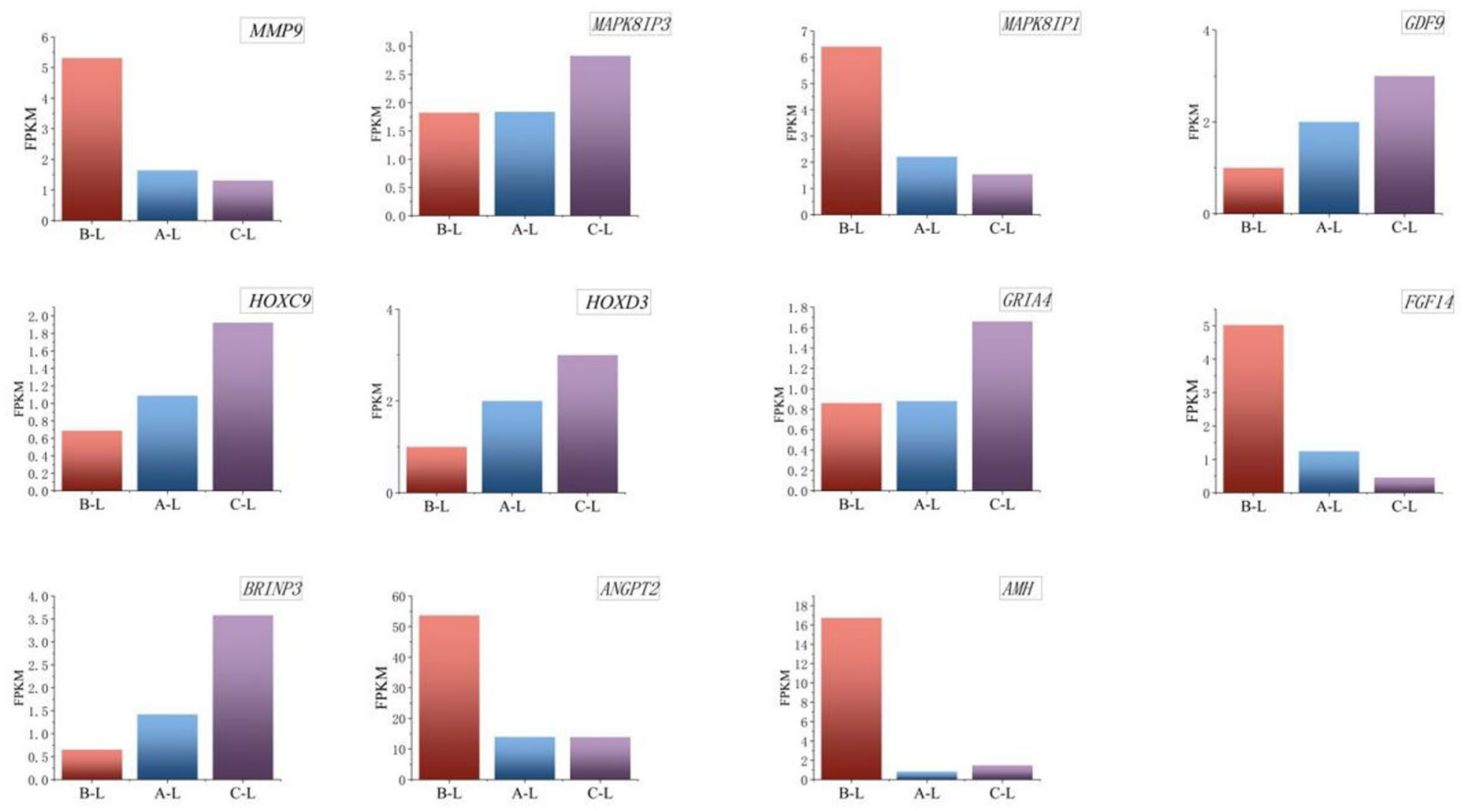
Expression levels of key differential mRNAs. From left to right are arranged in the order of *FecB^++^* genotype Pishan Red sheep, *FecB^B+^* genotype Red sheep, and Hu sheep. The *Y*-axis represents FPKM, a standardized metric for quantifying gene expression levels.

Transcriptomic profiling at the mRNA level identified critical genes – including *GDF9*, *GRIA4*, *HOXC9*, *HOXD3*, *MAPK8IP3*, *AMH*, *ANGPT2*, *FGF14*, *MAPK8IP1*, *MMP9*, and *BRINP3* demonstrating multifaceted roles in ovarian folliculogenesis, cell cycle modulation, and MAPK-mediated signal transduction cascades. Bar graph analysis of mRNA expression levels (Figure 8) revealed significant differences in gene expression across breeds, with these transcriptional disparities potentially linked to variations in their reproductive performance.

The *AMH* gene exhibited significantly higher expression levels in *FecB^++^*-genotype Pishan Red Sheep (Group B) compared to *FecB^B+^*-genotype (Group A) and Hu Sheep (Group C). This transcriptional pattern suggests that AMH may play a more prominent role in follicular quiescence regulation within mono-ovulatory breeds, while its activity in poly-ovulatory breeds is likely modulated through complementary regulatory mechanisms. The *ANGPT2* gene displayed the highest expression levels in *FecB^++^*-genotype Pishan Red Sheep, compared to *FecB^B+^*-genotype and Hu Sheep. This expression pattern may correlate with its dual roles in modulating ovarian angiogenesis and regulating follicular maturation during reproductive cycles. The *FGF14* gene exhibited the highest expression levels in *FecB^++^*-genotype Pishan Red Sheep, whereas significantly lower expression was observed in *FecB^B+^*-genotype and Hu Sheep. This differential expression pattern may be associated with FGF14’s regulatory functions in cellular proliferation and differentiation. The *HOXC9* and *HOXD3* genes exhibited significantly lower expression levels in *FecB^++^*-genotype Pishan Red Sheep compared to other genotypes, suggesting potential associations with their regulatory roles in embryonic morphogenesis and cellular differentiation processes. *MAPK8IP1* and *MMP9* exhibited the highest expression levels in *FecB^++^*-genotype Pishan Red Sheep, potentially linked to their functional roles in follicular maturation, ovulation dynamics, and extracellular matrix remodeling during ovarian tissue homeostasis.

**Figure 9:**
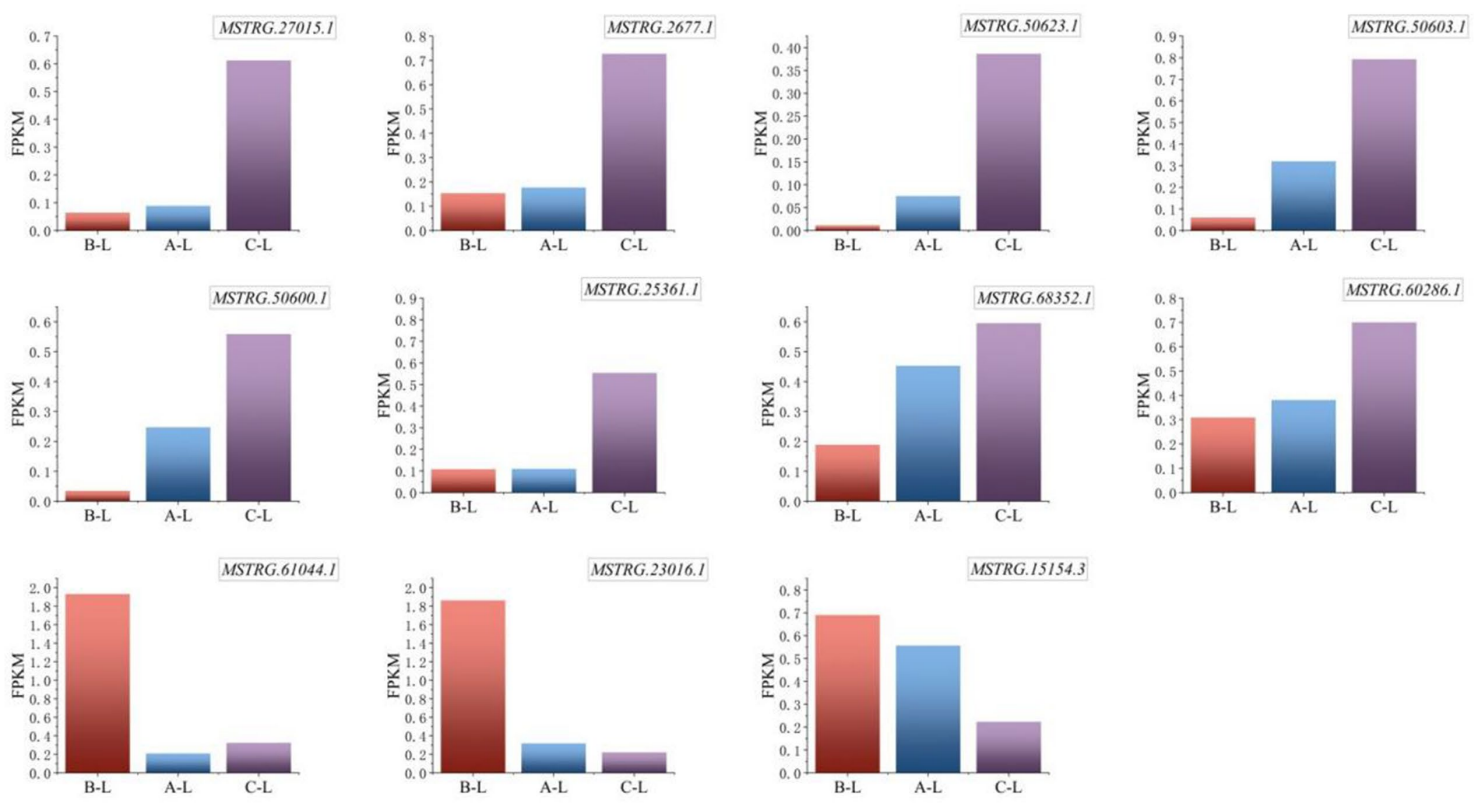
Expression levels of key differential lncRNAs. From left to right are arranged in the order of *FecB^++^* genotype Pishan Red sheep, *FecB^B+^* genotype Red sheep, and Hu sheep. The *Y*-axis represents FPKM, a standardized metric for quantifying gene expression levels.

Critical lncRNAs identified through systematic analysis include *MSTRG.61044.1*, *MSTRG.2677.1*, *MSTRG.23016.1*, *MSTRG.27015.1*, *MSTRG.60286.1*, and *MSTRG.15154.3*, which exhibited conserved expression patterns across genotype groups. The results (Figure 9) demonstrated significant differential expression of these lncRNAs across ovine breeds, a transcriptional divergence potentially associated with their reproductive performance variations.

Specifically, the lncRNA *MSTRG.61044.1*, which is highly expressed in *FecB^++^*-genotype Pishan Red Sheep, demonstrates dual regulatory mechanisms: it exerts cis-regulatory control over the adjacent *MAPK8IP1* gene, while trans-regulating reproduction-associated genes such as *AMH* and *CCL25*. These findings implicate *MSTRG.61044.1* as a potential hub regulator orchestrating follicular selection and gonadotropin-responsive signaling cascades. Further investigation revealed that *MSTRG.23016.1*, also highly expressed in *FecB^++^*-genotype Pishan Red Sheep, exhibits functional modulation of *MMP9* and *ANGPT2* transcripts, suggesting its potential involvement in extracellular matrix remodeling and angiogenic niche regulation during ovarian tissue homeostasis. Simultaneously, *MSTRG.15154.3* dynamically regulates granulosa cell proliferation and lineage commitment through cis-mediated transcriptional control of *ERBB4*, establishing a mechanistic link between lncRNA-driven chromatin interactions and follicular developmental competence. In contrast, Hu Sheep-specific highly expressed lncRNAs exhibited distinct functional orientations: *MSTRG.2677.1* modulates ovarian stromal cell homeostasis through trans-regulation of *HGF* and *BRINP3*, potentially orchestrating extracellular matrix dynamics and stromal-epithelial crosstalk during follicular wave progression. *MSTRG.27015.1* and *MSTRG.60286.1* were found to target *MAPK8IP3* and *PPP3CB*, respectively. This mechanistic association suggests their potential roles in regulating cell cycle checkpoints and orchestrating oocyte maturation through calcium-dependent signaling cascades. Notably, MAPK signaling pathway-associated genes (*MAPK8IP1* and *MAPK8IP3*) exhibit breed-specific differential regulation by distinct lncRNAs, which may reflect evolutionary divergence in follicular development synchronization mechanisms across ovine breeds.

### 3.10 Key mRNA-lncRNA correlation analysis

**Figure 10:**
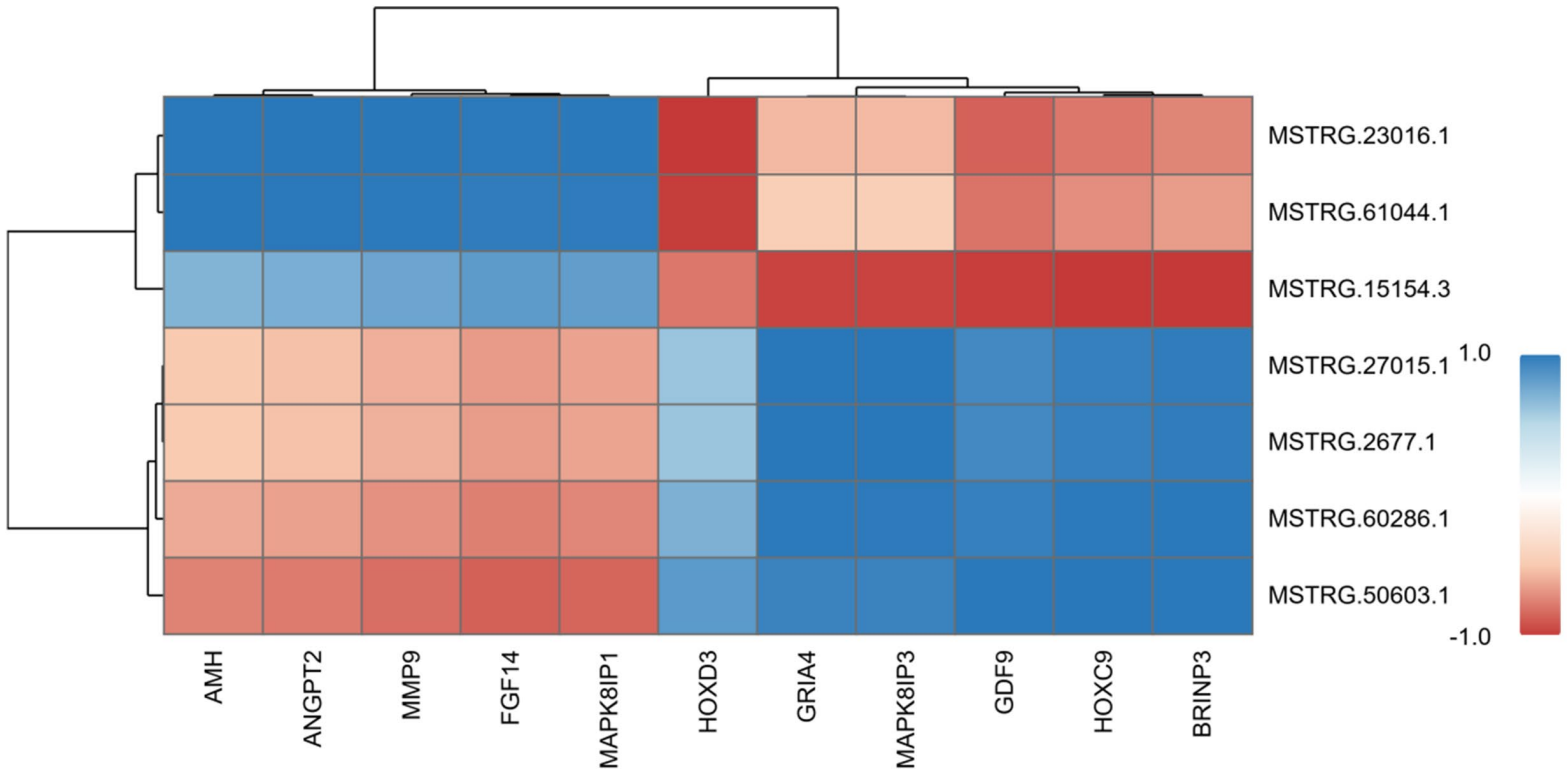
Hierarchical clustering heatmap of mRNA and lncRNA correlation analysis. In the heatmap, the numerical value within each colored block represents the correlation coefficient between the two samples aligned with the block’s position on the *X*-axis and *Y*-axis, where higher values denote stronger correlations. Red indicates positive correlation (maximum +1), and blue indicates negative correlation (minimum −1).

Figure 10 demonstrates the correlation heatmap between key differentially expressed mRNAs and lncRNAs identified in this study. The heatmap revealed strong correlations between key differentially expressed mRNAs and lncRNAs, with these RNA molecules distinctly clustering into two groups based on their expression correlation patterns. Specifically, lncRNAs *MSTRG.23016.1* and *MSTRG.61044.1* demonstrated strong positive correlations with key reproductive regulators including *AMH*, *ANGPT2*, *MMP9*, *FGF14*, and *MAPK8IP1*, while exhibiting robust negative correlations with the *HOXD3* gene. Furthermore, lncRNAs *MSTRG.2677.1*, *MSTRG.27015.1*, *MSTRG.60286.1*, and *MSTRG.50603.1* exhibited strong positive correlations with *GRIA4*, *MAPK8IP3*, *GDF9*, *HOXC9*, and *BRINP3* mRNAs. Notably, lncRNA *MSTRG.50603.1* also exhibited strong negative correlations with *MAPK8IP1*, *FGF14*, and *MMP9* mRNAs. Furthermore, *MSTRG.15154.3* displayed strong positive correlations with *FGF14* and *MAPK8IP1*, while showing negative correlations with *GRIA4*, *MAPK8IP3*, *GDF9*, *HOXC9*, and *BRINP3*. Such correlation-based co-expression analysis has proven instrumental in pinpointing putative functional interactions within RNA regulatory networks, holding significant implications for deciphering transcriptional circuitry and elucidating molecular mechanisms governing ovarian folliculogenesis. These findings provide critical leads for subsequent functional validation studies, facilitating in-depth exploration of the intricate interplay between mRNAs and lncRNAs, as well as their roles in disease pathogenesis and progression.

### 3.11 Real-time quantitative PCR (RT-qPCR) validation

**Figure 11:**
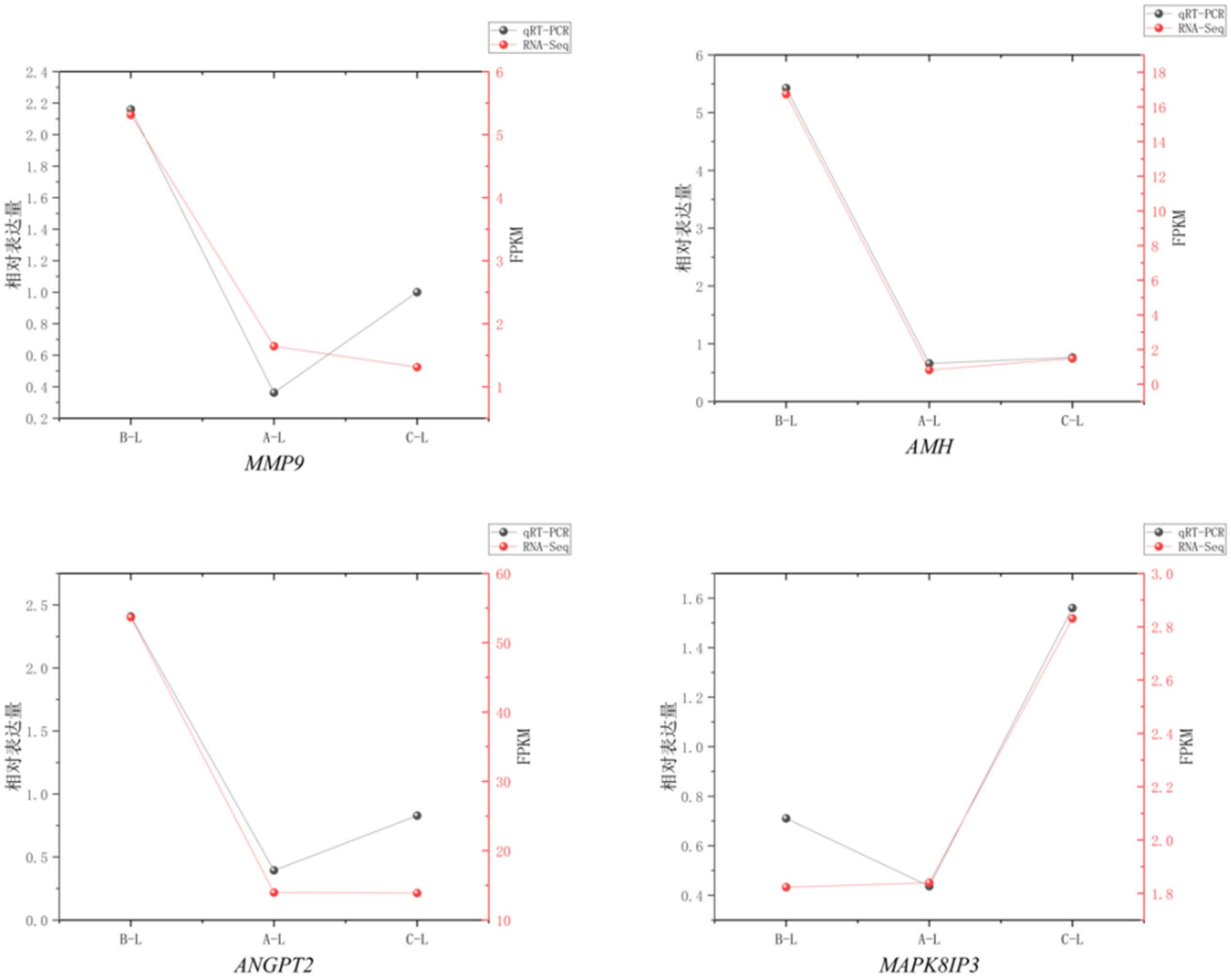
mRNA Real-time fluorescence quantitative PCR validation of RNA-Seq. Comparison of gene expression levels measured by qRT-PCR (black circles) and RNA-Seq (red squares). The *X*-axis represents different sample groups, arranged from left to right in the following order: *FecB^++^* genotype Pishan Red sheep, *FecB^B+^* genotype Red sheep, and Hu sheep. The left *Y*-axis indicates the relative expression level (qRT-PCR), while the right y-axis shows the RNA-Seq-derived FPKM values.

**Figure 12:**
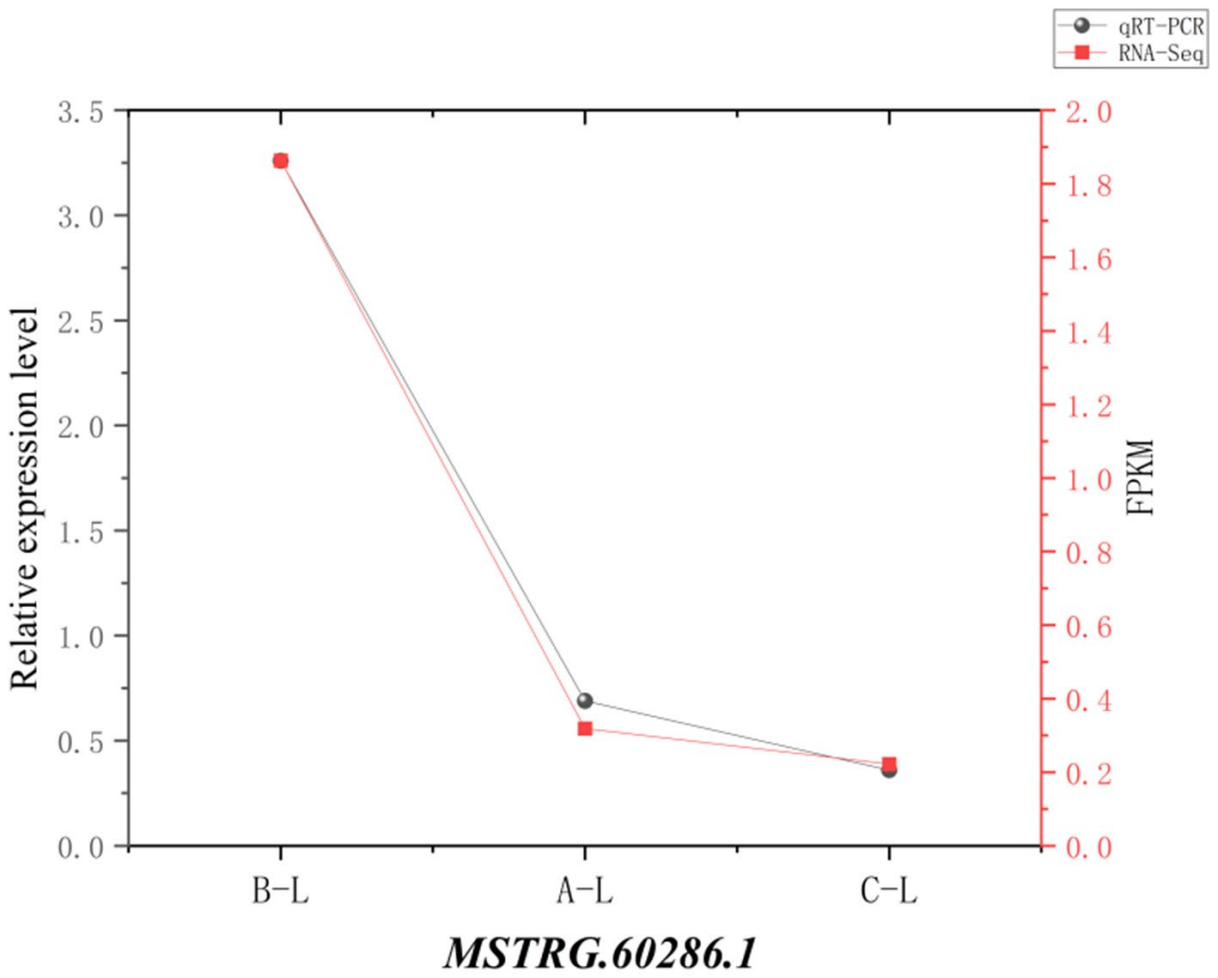
lncRNA real-time fluorescence quantitative PCR validation of RNA-Seq. Comparison of gene expression levels measured by qRT-PCR (black circles) and RNA-Seq (red squares). The *X*-axis represents different sample groups, arranged from left to right in the following order: *FecB^++^* genotype Pishan Red sheep, *FecB^B+^* genotype Red sheep, and Hu sheep. The left *Y*-axis indicates the relative expression level (qRT-PCR), while the right y-axis shows the RNA-Seq-derived FPKM values.

To assess the reliability of RNA sequencing, we randomly selected 4 mRNAs (*MMP9*, *AMH*, *ANGPT2*, *MAPK8IP3*) and 1 lncRNA (*MSTRG.60286.1*) for qRT-PCR validation. The results demonstrated consistent expression patterns across different reproductive stages between qRT-PCR and RNA-Seq data (Figures 11, 12), confirming the robustness of our transcriptomic analysis.

## 4 Discussion

Research on ovine reproductive efficiency has revealed that elevated expression levels of genes such as *GDF9* and *BMP15* are intricately linked to follicular development, suggesting their potential as molecular targets for improving reproductive performance (21). In the present study, the highest expression level of the *GDF9* gene was observed in Hu Sheep, followed by *FecB^B+^* genotype Pishan Red Sheep, with the *FecB^++^* genotype Pishan Red Sheep exhibiting the lowest expression. This finding suggests a potential positive correlation between *GDF9* expression levels and ovine prolificacy, i.e., elevated expression may enhance fecundity traits. Xie et al. (22) discovered the pivotal role of the *ELOVL7* gene in lipid metabolism in caudal fat. In this study, the high expression of GDF9 in ovarian tissue suggests its potential as a core regulatory node in reproduction control. Although *ELOVL7* (lipid metabolism) and *GDF9* (reproduction regulation) operate in distinct functional contexts, both influence cell fate by modulating key signaling pathways. This provides cross-phenotype research insights for elucidating the multi-tissue synergistic regulatory mechanisms underlying complex traits in sheep. Previous studies have demonstrated significant breed-specific variations in *GDF9* expression levels among ovine populations, with strong correlations observed between elevated expression and increased ovulation rates as well as improved litter size (21). Fengyan et al. (23) demonstrated that polymorphisms in the *GDF9* gene are significantly associated with litter size in Luzhong Meat Sheep, further reinforcing its critical role in modulating ovine prolificacy. Specifically, specific mutation loci in the *GDF9* gene are associated with increased litter sizes, suggesting that its expression level significantly influences ovine reproductive performance (24). This consensus aligns with the observed differential *GDF9* expression levels among ovine breeds in our study, empirically supporting its critical regulatory role in governing prolificacy traits.

*MMP9* exhibits elevated expression levels in ovine ovarian tissues, with pronounced activity notably observed during follicular development (25). Elevated *MMP9* expression may facilitate follicular maturation and ovulation, thereby enhancing ovulation rates. Studies have revealed (26), that *MMP9* expression increases significantly during follicular maturation, with the highest levels observed in the theca and granulosa cells of well-developed mature follicles. These findings collectively indicate that *MMP9* likely plays a regulatory role in coordinating follicular development and ovulatory processes within ovine ovarian tissues, offering mechanistic insights into its contribution to reproductive efficiency. Studies have demonstrated that *MMP9* deficient mice exhibit impaired embryonic development and dysregulated maternal-fetal interactions, characterized by intrauterine growth retardation and reduced litter size (27). This evidence strongly suggests that *MMP9* exerts a critical regulatory influence on trophoblast cell differentiation and placental maturation, with functional perturbations in these processes directly contributing to compromised reproductive outcomes. Tight regulation of *MMP9* expression is indispensable for maintaining physiological homeostasis, while dysregulated expression may be etiologically linked to reproductive pathologies such as impaired folliculogenesis and pregnancy disorders. *MMP9* participates in uterine remodeling through extracellular matrix degradation, thereby influencing embryo implantation and pregnancy maintenance (28).

The elevated expression of *BRINP3* in ovine ovarian tissues and its marked differential expression in prolific sheep suggest its potential functional significance in mediating key reproductive processes. *BRINP3* may modulate the expression of gonadotropins, thereby regulating follicular development and ovulation. The elevated expression of *AMH* in ovine ovarian tissues and its pivotal role in follicular development suggest its potential critical function in regulating reproductive processes in sheep. Experimental results demonstrate that *AMH*-deficient mice exhibit significantly accelerated follicular depletion, providing direct evidence for the functional association between *AMH* and reproductive lifespan regulation (29). This study elucidates for the first time that *AMH* maintains the dynamic equilibrium of follicular development through dual mechanisms: inhibiting primordial follicle recruitment and modulating follicle-stimulating hormone (FSH) receptor expression in granulosa cells. The establishment of an *AMH*-FSH-Inhibin B tripartite feedback regulatory system demonstrated that *AMH* suppresses FSH receptor expression in granulosa cells (with a 40% downregulation observed *in vitro*), thereby extending the survival duration of small antral follicles and augmenting ovulation potential (30). Qiang et al. (31) investigated the association between *AMH* genetic variants and reproductive performance in Lacaune sheep, identifying several single nucleotide polymorphisms (SNPs) significantly correlated with litter size. Hox genes, a highly conserved subgroup within the homeobox superfamily, play pivotal roles in developmental processes (32). Specifically, *HOXC9* and *HOXD3* are primarily responsible for orchestrating anterior–posterior axis formation and participate in the morphogenesis of specialized organ systems (33). Their expression typically exhibits marked temporal and spatial specificity, exerting profound regulatory effects on both the development and functional homeostasis of the reproductive system. Investigations into the expression patterns and functional roles of these genes provide critical insights into their potential regulatory mechanisms within ovine reproductive biology.

Studies have increasingly demonstrated that lncRNAs play indispensable roles in biological life cycles and reproductive processes (34, 35). Studies have revealed that lncRNAs are deeply involved in multiple reproductive processes, including ovarian development (36), oogenesis (37), and pregnancy maintenance (38), providing critical opportunities to investigate lncRNA-mediated regulation of ovine reproductive performance. In this study, comparative analysis of lncRNA and mRNA expression profiles in ovarian tissues during estrus between Pishan Red Sheep and Hu Sheep successfully identified key candidate mRNAs and their potentially regulatory lncRNAs. These findings further substantiate the pivotal role of lncRNAs in ovine reproduction.

lncRNAs regulate mRNA expression through both cis- and trans-acting mechanisms. Cis-acting lncRNAs modulate target gene expression through enhancer-like mechanisms, either activating or repressing transcription (39). Trans-acting lncRNAs regulate mRNA splicing, stability, and translation by interacting with proteins, DNA, and other RNAs (40). Xiaojing et al. (41) demonstrated that lipopolysaccharide (LPS) treatment induces significant alterations in the expression profiles of both lncRNAs and messenger RNAs (mRNAs) in bovine mammary epithelial cells. Zhang et al. (42) conducted RNA sequencing analysis of hypothalamic lncRNAs in Small Tail Han sheep carrying the *FecB^++^* genotype, identifying potential reproductive regulators such as *MSTRG.26777* and *MSTRG.105228*. These findings provide novel insights into the hypothalamic regulation of ovine reproduction. The lncRNAs and mRNAs associated with ovine fecundity identified in our study corroborate the findings of Zhuangbiao Zhang et al., providing further evidence for the critical role of lncRNAs in sheep reproduction. Shabbir et al. (11) systematically characterized stage-specific lncRNA and mRNA expression profiles in Hu Sheep ovaries across follicular developmental stages, identifying differentially expressed miRNAs and lncRNAs that form potential regulatory networks. The identified genes are functionally implicated in ovarian follicular development and steroid hormone-mediated signaling pathways, among other critical reproductive processes. These findings align with the roles of lncRNAs identified in our study—regulating follicular selection, hormonal response, ovarian tissue remodeling, and cellular proliferation/differentiation—collectively advancing the mechanistic understanding of ovine reproduction. However, functional validation of specific candidate genes remains warranted.

## 5 Conclusion

This study elucidates the molecular determinants of ovine reproductive efficiency through systematic analysis of differential expression profiles of lncRNAs and mRNAs in ovarian tissues during the estrous cycle between Pishan Red Sheep and Hu Sheep, unraveling key genetic regulators and their multifaceted mechanisms in follicular development and ovulation competency. A suite of key mRNAs, including *GDF9*, *GRIA4*, *HOXC9*, *HOXD3*, *MAPK8IP3*, *AMH*, *ANGPT2*, *FGF14*, *MAPK8IP1*, *MMP9*, and *BRINP3*, was successfully identified through systematic transcriptomic screening. Furthermore, we identified lncRNAs with regulatory interactions to these mRNAs, including *MSTRG.61044.1*, *MSTRG.23016.1*, *MSTRG.15154.3*, *MSTRG.2677.1*, *MSTRG.27015.1*, and *MSTRG.60286.1*. These lncRNAs exert critical regulatory roles in follicular selection, hormone responsiveness, ovarian tissue remodeling, and cellular proliferation/differentiation through coordinated cis- and trans- regulatory mechanisms. The findings of this study establish a molecular framework for refining ovine reproductive management strategies and pinpoint critical gene regulatory hubs that may serve as pivotal modulators of reproductive efficiency.

## Data Availability

The RNA-Seq data of Pishan Red Sheep and Hu Sheep have been deposited in the Sequence Read Archive (SRA) database of the National Center for Biotechnology Information (NCBI) under BioProject accession number PRJNA1226111.

## Footnotes

^1^ www.biocloud.net

## Supplementary material

SUPPLEMENTARY TABLE 1 Top five upregulated and downregulated mRNAs in estrus ovarian tissues.

SUPPLEMENTARY TABLE 2 Top five upregulated and downregulated lncRNAs in estrus ovarian tissues.
