# Supplementary material for: Differential expression analysis of lncRNA and mRNA in ovarian tissues of Pishan Red Sheep and Hu Sheep with distinct genotypes during estrus
Source: Front Vet Sci. 2025 Sep 24;12:1614599. doi: 10.3389/fvets.2025.1614599 (PMC12504091; doi:10.3389/fvets.2025.1614599)
Supplement: SUPPLEMENTARY TABLE 1 — Top five upregulated and downregulated mRNAs in estrus ovarian tissues. [file Supplementary_Tables.docx]

**Supplementary Table 1: Top five upregulated and downregulated mRNAs in estrus ovarian tissues**

| differentially expressed group | #ID | Pvalue | log2FC | regulated |
| --- | --- | --- | --- | --- |
| A-LvsB-L | LOC101107030 | 0.000253342328671836 | 5.33780660582726 | up |
|  | AMH | 0.00353166523542848 | 4.25783582438397 | up |
|  | IHH | 0.000109715902369519 | 3.56641480814088 | up |
|  | HSD17B1 | 0.000102926774203361 | 3.3870070366338 | up |
|  | CLEC2L | 0.00232616263339318 | 3.20047691356932 | up |
|  | LOC101114790 | 0.00027198025081264 | -6.563228367 | down |
|  | IL1RL1 | 0.00149232539649362 | -6.163202711 | down |
|  | STAR | 0.0014941862540478 | -5.8246189 | down |
|  | HSD3B1 | 0.00094263092879345 | -5.811930407 | down |
|  | PTHLH | 0.000177642393885209 | -5.019624565 | down |
| A-LvsC-L | LOC101107030 | 0.000846132678401608 | 6.36284544496467 | up |
|  | PCDH11X | 3.52366218895884e-13 | 5.47435972771716 | up |
|  | FCAR | 2.13728371386504e-08 | 5.19092793580896 | up |
|  | GPNMB | 5.91238175409151e-08 | 4.95883353979888 | up |
|  | PRG4 | 7.80694079660879e-05 | 4.35901108466009 | up |
|  | OVGP1 | 3.46313777872141e-08 | -10.75044822 | down |
|  | DNAH12 | 6.30306157000878e-06 | -8.033363598 | down |
|  | DNAH5 | 1.61317345390699e-05 | -7.674473206 | down |
|  | EHF | 3.1572410179693e-05 | -7.613209155 | down |
|  | TEKT1 | 0.000104301210468841 | -6.98209533 | down |
| B-LvsC-L | TNFAIP6 | 1.29484577066895e-07 | 5.42988095657133 | up |
|  | PCDH11X | 7.37157179196282e-27 | 4.33427987390359 | up |
|  | LYZ | 7.57110644241685e-12 | 3.86680823498454 | up |
|  | LOC101111409 | 7.36379575836463e-12 | 3.66600586173498 | up |
|  | DQA | 7.28170209609294e-07 | 3.60858180121631 | up |
|  | ADGB | 4.55264584324396e-18 | -9.544536351 | down |
|  | FOXJ1 | 6.00314305583188e-10 | -9.908732943 | down |
|  | RIBC2 | 8.59387967066165e-14 | -9.961920037 | down |
|  | APOBEC4 | 2.68154780708022e-14 | -10.69839932 | down |
|  | CAPSL | 2.96856538156343e-22 | -10.79336966 | down |

**Supplementary Table 2: Top five upregulated and downregulated lncRNAs in estrus ovarian tissues**

| differentially expressed group | #ID | Pvalue | log2FC | regulated |
| --- | --- | --- | --- | --- |
| A-LvsB-L | MSTRG.88405.1 | 6.57270503788309e-09 | 7.19850314575203 | up |
|  | MSTRG.55203.3 | 0.00101751534010199 | 7.16299960445698 | up |
|  | MSTRG.43500.1 | 3.26088297517409e-08 | 6.40393188246086 | up |
|  | MSTRG.19237.4 | 2.63226050771537e-06 | 6.39503160163919 | up |
|  | MSTRG.39351.6 | 4.6465855217163e-08 | 6.33230627890888 | up |
|  | MSTRG.6569.54 | 1.88925849040187e-16 | -9.99320203 | down |
|  | MSTRG.46101.17 | 1.04018452675485e-08 | -8.810159204 | down |
|  | MSTRG.14289.5 | 3.98541851048476e-12 | -8.674645961 | down |
|  | MSTRG.24616.2 | 2.47961078737623e-11 | -8.068071244 | down |
|  | MSTRG.70425.5 | 1.24340448803897e-08 | -7.789267608 | down |
| A-LvsC-L | MSTRG.52728.9 | 4.60047857160037e-13 | 9.06040233031223 | up |
|  | MSTRG.66214.15 | 1.38384973930499e-14 | 8.79311280633395 | up |
|  | MSTRG.5913.3 | 1.17403383530407e-09 | 8.52669138395705 | up |
|  | MSTRG.48879.5 | 2.71437698824431e-11 | 8.37608611139312 | up |
|  | MSTRG.80042.6 | 3.44790241867031e-11 | 8.0855761614962 | up |
|  | MSTRG.82423.3 | 3.5445768443829e-07 | -7.451165898 | down |
|  | MSTRG.28576.1 | 4.1751937054258e-11 | -8.81233759 | down |
|  | MSTRG.24616.2 | 2.86140836961386e-14 | -9.274216147 | down |
|  | MSTRG.80614.2 | 9.0264674850722e-14 | -9.352844359 | down |
|  | MSTRG.32744.3 | 6.27206051510646e-16 | -10.30487806 | down |
| B-LvsC-L | MSTRG.3221.3 | 3.01580991791458e-15 | 9.06227217644675 | up |
|  | MSTRG.52728.9 | 2.96116778885647e-13 | 9.01287018892882 | up |
|  | MSTRG.5913.3 | 8.85847085064349e-10 | 8.47869737042864 | up |
|  | MSTRG.45513.2 | 4.07983590456998e-11 | 8.42766083414024 | up |
|  | MSTRG.32423.2 | 9.91585262568183e-19 | 8.24491529506198 | up |
|  | MSTRG.55203.3 | 1.03144319889155e-49 | -16.41266679 | down |
|  | MSTRG.55203.5 | 2.61797587608568e-43 | -15.27169605 | down |
|  | MSTRG.47482.13 | 3.98760326351698e-11 | -10.43687069 | down |
|  | MSTRG.15681.10 | 1.94868283824793e-10 | -9.490972205 | down |
|  | MSTRG.88161.3 | 1.04128725062018e-13 | -9.460617562 | down |
